# Remarks on Hypochondriasis, Melancholia, and Insanity

**Published:** 1829-10-01

**Authors:** 


### XXXV.

### MADRAS MILITARY HOSPITAL.

REMARKS ON HYPOCHONDRIASIS, MELANCHOLIA, AND INSANITY. By JAMES ANNESLEY, Esq.

In the second volume of Mr. Annesley's work on Indian Diseases, that zealous observer has made some remarks on the influence of morbid secretions in the bowels upon the mental faculties. Adverting to the statement made by Esquirol, (but which, by the bye, has not been corroborated by the experience of others) respecting the displacement of the colon in 33 out of 168 cases of insanity, Mr. A. observes :—

"Now whether this derangement proceed from accumulations of fæcal matters in this viscus, or from great relaxation and want of tone of its coats and peritoneal covering, the necessary consequence of this condition must have been, in every case, to have favoured an unnatural retention and collection of fæcal and excrementitious matters in the bowel, and to have generated disease in the mucous surface on which they lodged, inducing sympathetic disorder in various parts of the system, more particularly in the brain itself."

Without pretending that this may be a general, or even a frequent cause of mental derangement, Mr. A. very properly directs the attention of his brethren to the subject of purgation in mania and melancholia. In this he is supported by the ancients, who were good observers, though bad theorists. They, as is well known, employed the most active vegetable cathartics, especially hellebore, conceiving that the removal of black bile would be the removal of the disease.

"In a very great proportion of cases of melancholic alienation of mind, and indeed in other forms of mental disorder the stools procured by means of active purging are very dark, tenacious, and even of a pitchy blackness, resulting, in our opinion, from the accumulation and admixture of the various secretions, excrementitious matters, and imperfectly digested food, poured into the alimentary canal, and from the changes they have undergone during their retention in that situation."

With this view our author has always made it a point, in cases of mental alienation, to institute a most active and uninterrupted course of purgative medicines in conjunction with those means which act in restraining the activity of circulation in the brain. Thus purgative medicines have been daily prescribed for months in succession, while general or local blood-letting has also been frequently employed. Mr. A. has occasionally exhibited tonics and cordials during this course of depletion and purgation, with the view of supporting the energies of life, and preventing exhaustion.

Melancholia and mania frequently occur among Europeans in hot climates, and Mr. A. informs us that he has had extended experience in those complaints. According to his experience, morbid accumulations in the bowels formed one of the characteristics of this class of maladies, while the energetic employment of purgatives always proved decidedly successful in the treatment. Mr. A. has detailed several cases verbatim from the hospital reports.

"In all of them, the employment of purgatives brought away most abundant motions of a very dark, tenacious, and offensive description. In some of those cases, the purgative plan had been continued for many days and even for several weeks before it succeeded in detaching the morbid secretions adhering to the coats of the bowels; but, in every case, disorder began to yield as soon as these were carried off, and the motions to assume a healthy character."

Mr. A. cautions us against intermitting the purgatives even for a single day, till the secretions become healthy, *after* the evacuation of motions similar to the above. The mere colour of the fæces is not to be trusted.

"They are often very offensive, of a peculiarly disagreeable odour, and when minutely examined, as they always ought to be, they are observed to be tenacious, like bird-lime, of a putty-like consistence, and streaked with various shades of colour, even when reported to the practitioner as being of a natural appearance. The minute examination of the alvine evacuations is a point of the utmost importance in practice, more especially in the disorders under consideration; and although it is not an agreeable office, is a very necessary one, and will be justly considered as such by those who wish to treat disease successfully. It cannot, therefore, be too strongly impressed upon the mind of the inexperienced practitioner."

This inspection of the excretions is too often neglected in private practice, to the great detriment of the patient and the disappointment of the physician. We shall glance at one or two cases.

*Case* 1. Colonel ———, had been affected for some years with dyspeptic complaints, accompanied by attacks of calculous affections, during which there was great depression of spirits amounting to melancholia. Mr. A. prescribed aloetic and mercurial pills at night, and bitter aperients in the morning. These medicines had an excellent effect and procured one or two copious motions daily.

"The discharge from the bowels was at first extremely morbid, black, tenacious, and offensive, and, so long as they continued in that state, the laxative was pursued without intermission. As the alvine discharges became more natural and healthy, his general health improved, but he suffered occasionally from extremely low spirits and sinking, often followed by general coldness over his whole body; and whenever this took place, the motions were always black, adhesive, and tenacious, like tar, the removal of which from the coats of the intestines appeared to cause this sense of sinking and coldness. As a temporary measure, the camphor mixture with aqua ammoniæ and spiritus ætheris vitriolici was then used, and with advantage; but he always improved and got better after the black, tar-like motions were removed. About three weeks after he first put himself under our care, we had recourse to the nitro-muriatic solution, and the body was sponged morning and evening with it. He continued this remedy whilst he was under our care with manifest benefit; the functions of the liver became more active, and the bile was secreted of a more healthy quality; the bowels performed their duty more regularly, though he was obliged occasionally to have recourse both to the pills and mixture, which we still recommended him to continue."

*Case* 2. Mental Alienation, with great Timidity.— Lieut. ———, was admitted. It was stated that mental derangement came suddenly on while proceeding by sea to Quilon. He fancied himself guilty of some great crime which he would not explain, and that very one he sees has a design against his life. Pulse soft and full—skin rather hot—face flushed—tongue coated yellow—bowels constipated. A brisk purgative, consisting of ten grains of calomel, followed by the senna draught, was exhibited, and produced copious discharges by vomiting and stool. Next day he said he felt better, but was still labouring under mental aberration, having suffused eyes, contracted pupils, and heat about the head. Leeches, blisters, purgation. The dejections on the third day were copious, black, and very fetid. The aberration continued, and the purgative plan was persevered in. At the end of the third week he was discharged cured.

*Case* 3. The wife of one of the soldiers was admitted into hospital in a state of mental derangement, with all the usual symptoms, she being then in the fifth month of pregnancy. She was put upon a plan of purgation with local depletion, as in the preceding case, and recovered in little more than a fortnight. In the course of the treatment, great quantities of scybalous and other morbid matters came away.

We need not detail any more cases, as the methodus medendi was nearly the same in all, however varied were the phenomena of hallucination. There can be no doubt, indeed, that purgation is one of the most important items in the catalogue of remedies for mental alienation, dependent, as it so often is, on gastric and intestinal disorder.

---

